# A closer look at cognitive control: differences in resource allocation during updating, inhibition and switching as revealed by pupillometry

**DOI:** 10.3389/fnhum.2015.00494

**Published:** 2015-09-10

**Authors:** Eefje W. M. Rondeel, Henk van Steenbergen, Rob W. Holland, Ad van Knippenberg

**Affiliations:** ^1^Thales Research and TechnologyDelft, Netherlands; ^2^Behavioural Science Institute, Radboud UniversityNijmegen, Netherlands; ^3^Leiden Institute for Brain and Cognition, Leiden UniversityLeiden, Netherlands; ^4^Institute of Psychology, Leiden UniversityLeiden, Netherlands

**Keywords:** cognitive control, pupil dilation, resource allocation, updating, switching, inhibition

## Abstract

The present study investigated resource allocation, as measured by pupil dilation, in tasks measuring updating (2-Back task), inhibition (Stroop task) and switching (Number Switch task). Because each cognitive control component has unique characteristics, differences in patterns of resource allocation were expected. Pupil and behavioral data from 35 participants were analyzed. In the 2-Back task (requiring correct matching of current stimulus identity at trial p with the stimulus two trials back, p −2) we found that better performance (low total of errors made in the task) was positively correlated to the mean pupil dilation during correctly responding to targets. In the Stroop task, pupil dilation on incongruent trials was higher than those on congruent trials. Incongruent vs. congruent trial pupil dilation differences were positively related to reaction time differences between incongruent and congruent trials. Furthermore, on congruent Stroop trials, pupil dilation was negatively related to reaction times, presumably because more effort allocation paid off in terms of faster responses. In addition, pupil dilation on correctly-responded-to congruent trials predicted a weaker Stroop interference effect in terms of errors, probably because pupil dilation on congruent trials were diagnostic of task motivation, resulting in better performance. In the Number Switch task we found higher pupil dilation in switch as compared to non-switch trials. On the Number Switch task, pupil dilation was not related to performance. We also explored error-related pupil dilation in all tasks. The results provide new insights in the diversity of the cognitive control components in terms of resource allocation as a function of individual differences, task difficulty and error processing.

## Introduction

In most new and complex situations people need cognitive control to behave in an adaptive manner. Cognitive control, often linked to the prefrontal cortex of the brain, comprises a set of cognitive processes that serve goal-directed behavior (e.g., Posner and Snyder, 1975; Cohen, 2001). Older literature described cognitive control as the ability to overcome strongly activated response tendencies (see e.g., Posner and Snyder, 1975), but more recently, the processes involved in cognitive control have been found to be more diverse (see e.g., Smith and Jonides, 1999). Miyake et al. (2000) explicitly addressed the unity and diversity of these processes and proposed three distinct components of cognitive control: updating (or monitoring), inhibition and switching (or shifting). Presumably, these components of cognitive control are based on different interactions between the prefrontal and the basal ganglia (Miyake and Friedman, 2012).

*Updating* refers to the ability to monitor and encode new information and replace old, no longer relevant information. A typical task to measure updating abilities is the so-called n-Back task (Kirchner, 1958). In an n-Back task, participants have to indicate whether a stimulus that is presented on the screen is the same or different as the stimulus presented *n* trials back. Thus, the task requires repeated updating of working memory. In the present study we used a 2-Back task. *Inhibition* refers to the ability to inhibit dominant, automatic or prepotent reactions when needed. A widely used task to measure response inhibition is the Stroop task (Stroop, 1935). In the Stroop task, people have to indicate the color of the ink in which a word is printed, while ignoring the meaning of the word. In congruent trials, the color of the ink is the same as the meaning of the word (e.g., “blue” printed in blue). In incongruent trials, the color of the ink is different from the meaning of the word (e.g., “blue” printed in yellow). The latter elicits a conflict and requires inhibition of the meaning of the word in order to respond correctly. Finally, *switching* refers to the ability to disengage from task sets and actively engage in new task sets (Monsell, 1996). In a typical switching paradigm, people have to respond to stimuli, e.g., numbers from one to ten, and use different rules for responding to these stimuli depending on a specific cue, e.g., the color of the stimulus. For example, when the number is printed in yellow, participants have to indicate whether it is odd or even. When the number is printed in blue, participants have to indicate whether it is greater than five, or smaller than/equal to five (Monsell et al., 2003). Switching between these task rules from one trial to the next is cognitively engaging and requires cognitive control.

Because moderately positive correlations have been observed between the three different cognitive control components (e.g., Miyake et al., 2000; Figure 2, report inter-component correlations ranging from 0.42 to 0.62), they seem to represent related, but distinct constructs (Miyake et al., 2000). Consistent with this idea, the three cognitive control components each have a different predictive value for complex cognitive tasks. Performance on the Wisconsin Card Sorting task was found to be strongly related to measures tapping into switching, the Tower of Hanoi task to measures tapping into inhibition, and the Operation Span (OSPAN) task to measures tapping into updating (Miyake et al., 2000). These findings indicate that each cognitive control component has its own unique contribution to performance on complex cognitive tasks. Moreover, research on decision making suggests that the three cognitive control components predict decision making behavior in different ways. For example, Del Missier et al. (2012) found that inhibition and updating were related to resistance to framing effects, better application of decision rules and enhanced cognitive reflection, whereas switching was found to be related to risk perception. These findings provide supportive evidence for the diversity of the three cognitive control components.

### Pupil Dilation and Resource Allocation

One of the most important aspects of cognitive control is the adaptive up- and down-regulation of resources, likely supported by neural systems involved in the detection of the need for cognitive control, such as the anterior cingulate cortex (Botvinick et al., 2001). Resource allocation refers to the proportion of resources that is actually invested in cognitive processing (see e.g., Just et al., 2003). Differences in resource allocation are highly relevant, as the amount of resources that a person allocates to a cognitively complex task may be indicative of ability and plausibly related to performance (Just et al., 2003; van der Meer et al., 2010). Despite the crucial role of resource allocation in performance on cognitive control tasks, thus far, indicators of resource allocation have not been systematically linked to the various processes of cognitive control, i.e., updating, inhibition and switching. Studying resource allocation during tasks measuring these three cognitive control components may provide useful insights in further differences and similarities between these three components.

In the present study, pupil dilation measures were used to investigate the dynamics of resource allocation while keeping primary causal factors such as illumination constant. Pupils constrict with parasympathetic activity and dilate with sympathetic activity (Lowenstein and Loewenfeld, 1950; Steinhauer et al., 2004). The notion that pupil dilation is a useful indicator of resource allocation has been around for quite some time (Lowenstein and Loewenfeld, 1962; Hess and Polt, 1964; Kahneman and Beatty, 1966; Kahneman, 1973). In recent years, the idea that pupil dilation can be used as an unobtrusive online indicator of resource allocation is still widely supported (e.g., Just et al., 2003; Bijleveld et al., 2009; van der Meer et al., 2010; Ariel and Castel, 2014). Recently, an increasing number of studies have also used pupil dilation as an index for activity in the locus coeruleus-norepinephrine (LC-NE) system (e.g., Jepma and Nieuwenhuis, 2011; Murphy et al., 2011). Receiving input from regions signaling the need for cognitive control, such as the anterior cingulate cortex, the LC-NE system is thought to play an important role in the recruitment of prefrontal regions when cognitive resouces are needed (Aston-Jones and Cohen, 2005; Verguts and Notebaert, 2009). This system could potentially also modulate prefrontal-cortex basal-ganglia interactions that underly the different components of cognitive control (Miyake and Friedman, 2012).

Although the relationship between pupil dilation and resource allocation had received empirical support, changes in pupil size have also been linked to other constructs as well, such as arousal (e.g., Bradley et al., 2008), reward (e.g., Bijleveld et al., 2009) and affect (e.g., Partala and Surakka, 2003). These constructs, however, are often intrinsically related to resource allocation. For example, when a stimulus is rewarding, important or significant, people are more likely to allocate resources to process the stimulus (e.g., see Bijleveld et al., 2009; but cf. Baumeister, 1984).

To complicate matters, besides being indicative of resource allocation (effort, attention), other causal factors may also impact pupil dilation, such as making errors. On theoretical grounds, we distinguish three different sources of variance in pupil dilation during cognitive control tasks, i.e., (1) individual differences in resource allocation; (2) task difficulty effects on resource allocation; and (3) error related pupil dilation.

#### Individual Differences in Resource Allocation

First, an increase in resource allocation to a task is expected to result in better task performance. To the extent that pupil dilation is diagnostic of resource allocation, differences between participants in pupil dilation during the execution of cognitively demanding tasks reflect differences in resource allocation to these tasks, which are presumably positively related to performance. Studies on fluid intelligence support this idea. van der Meer et al. (2010) found that participants with high scores on a fluid intelligence task showed the same amount of pupil dilation as participants with low scores on a fluid intelligence task on a simple task. However, on a difficult task, higher intelligence scores were associated with larger pupil dilation and better performance.

These ideas have hardly been tested using measures of cognitive control. Some studies addressed pupil dilation in the n-Back task, as indicated above, a task measuring updating ability. Karatekin et al. (2007, 2009) addressed pupil dynamics in a spatial 0- and 1-back task. They found that pupil dilation was higher for 10-year olds than for adults and that pupil dilation was higher on a 1-back task compared to a 0-back task (on the 0-back task participants were instructed to correctly respond to the stimulus location on the computer screen). Moreover, illustrating individual differences in resource allocation, they found a positive correlation between pupil dilation during correct responses and overall performance on these updating tasks, but only for participants with attention deficit hyperactivity disorder (ADHD). A plausible explanation for the absence of a positive correlation for healthy controls is that the tasks in Karatekin et al.’s studies (a spatial 0- and 1-back task) were relatively easy for healthy participants. Pupil dilation might not be related to performance on very simple tasks because enhanced recruitment of resources is not necessary for optimal performance on such tasks (van der Meer et al., 2010; cf. Bijleveld et al., 2009).

#### Task Difficulty

Second, we propose that pupil dilation varies as a function of task difficulty or, within a task, a function of trial difficulty (e.g., Kahneman, 1973), which also involve prefrontal cortex modulation (Botvinick et al., 2001). In a classic study by Kahneman and Beatty (1966), using a short-term memory task, the link between task difficulty and pupil dilation was elegantly illustrated. In the study, people had to remember strings of 3, 4, 5, 6 or 7 digits, pupil dilation was highest when people had to remember 7-digit strings and lowest when people had to remember 3-digit strings. More recently, Bijleveld et al. (2009) showed, using pupillometry, that individuals allocate more resources when responses are potentially rewarding and difficult.

Effects of diffulty can also be obtained in cognitive control measures as a function of trial difficulty. For example, when people respond to a Stroop (or a Switching task), more resources might be recruited when responding to a -relatively difficult—incongruent trial (or a switch trial), compared to a—relatively easy—congruent trial (non-switch trial). Indeed, some studies have investigated pupil dilation in the Stroop task and obtained larger pupil dilation for incongruent stimuli than for congruent stimuli (see e.g., Brown et al., 1999; Laeng et al., 2011). Importantly, such trial-type-dependent resource allocation (and attendant pupil dilation) need not be positively related to performance (e.g., faster RTs, fewer errors) on the trials involved. Instead, here effort allocation may even be negatively related to performance on the trials involved due to its co-variation with trial difficulty. Indeed, in one of the studies employing pupillometry within a Stroop task it was found that a greater difference in pupil dilation between incongruent and congruent trials was related to a greater difference in reaction times between incongruent and congruent trials (Laeng et al., 2011). Thus, in this study, differential effort allocation to difficult and easy trials was negatively related to performance on these trials due to its co-variation with trial difficulty (cf. van Steenbergen and Band, 2013).

#### Error-Related Pupil Dilation

A third source of pupil dilation within cognitive control tasks is related to incorrect vs. correct responding. Previous research suggests that autonomic arousal is higher when an incorrect response is given, perhaps reflecting anterior cingulate activation (Hajcak et al., 2003; Critchley et al., 2005). Therefore, errors within cognitive control tasks could elicit higher pupil dilation.

### The Present Research

The findings described above already suggest particular patterns in pupil dynamics that are specific for different cognitive control tasks, related to task difficulty and individual differences in resource allocation (as reflected in pupil dilation). Yet, a systematic study of resource allocation by investigating pupil dynamics in all three cognitive control components has not yet been conducted.

In the present research, we investigated resource allocation by the use of pupil dynamics during three tasks that tap into updating, inhibition or switching. To measure updating, we administered an n-Back task. As the tasks used in Karatekin et al. (2007, 2009) might have been relatively easy for healthy controls, we administered a more difficult version of the n-Back task, namely a 2-Back task. To measure inhibition and switching we administered a Stroop task and a Number Switch task respectively.

The following hypotheses will be tested:

1. *Individual differences in resource allocation*. First, we will test whether between-participant differences in average pupil dilation are related to overall performance on the cognitive control tasks. Given the expected influence of trial difficulty within the Stroop Task and the Switching Task on performance and pupil dilation (see hypothesis 2), this first hypothesis is optimally tested within the 2-Back task. Higher pupil dilation is predicted to be related to better performance on this task (in terms of errors and average RT). We will also explore the role of pupil dilation during encoding (pupil dilation during trial p-2) and retrieval (pupil dilation during trial p) on performance. Finally, we will also test whether individual differences in pupil dilation predict performance for incongruent and congruent trials (within the Stroop task) and for switch and non-switch trials (within the Number Switch task) separately.
2. *Trial difficulty*. We hypothesized that within the Stroop task and the Number Switch task, pupil dilation when responding to incongruent trials and switch trials will be higher than pupil dilation when responding to congruent and non-switch trials, respectively, based on the assumption that the former trial type is more difficult than the latter trial type. Furthermore, we predict that this difficulty-related pupil dilation co-varies with the strength of the Stroop interference effect and the level of the switch costs in the Switching task.
3. *Error-related pupil dilation*. Additionally, in all cognitive control tasks we explored error-related resource allocation by investigating error-related pupil dilation. Error-related resource allocation might play a role in regulating cognitive control, perhaps because of the activation of parts of the anterior cingulate cortex. Investigating differences in error-related resource allocation in a switching, updating, and inhibition task might provide more insight into the differences in resource allocation in these tasks.

## Materials and Methods

### Participants

Forty-one students from Leiden University, 6 male and 35 female, participated in the study in exchange for money (7.50 euros). Participants indicated they were not colorblind and were not taking any prescribed medication. Ages ranged from 17 to 29, *M* = 20.98, *SD* = 2.81. For six participants, severe technical problems occurred during the experiment. For example, pupil dilation was not recorded for a large part of the experiment, loud noise was produced in the adjacent room, or the participant’s cellphone was not switched off and disturbed the experiment. These participants were not included in the analyses. The experiment was conducted with healthy human participants, and did not utilize any invasive techniques, substance administration or psychological manipulations. The experiment was conducted according to institutional guidelines and approved by the local ethics committee. The study was conducted, and written informed consent of each participant was obtained in compliance with the principles contained in the Declaration of Helsinki.

### Materials

To ensure equal luminance across all trials in the administered cognitive control tasks, we used isoluminant Teufel colors for all stimuli and backgrounds (Teufel and Wehrhahn, 2000). All inter-trial intervals were set at 2000 ms (see e.g., Karatekin et al., 2007). In all cognitive control tasks, participants received feedback in the practice trials but not in the experimental trials.

#### Updating Task

To measure updating, we used a 2-Back task (see Jonides et al., 1997). In this task, participants were shown letters consecutively presented on the screen for 1500 ms or until a response was given. Letter font was Courier New (RGB color of font 188, 175, 081), 50 pt. For each letter, participants had to indicate whether the letter on the screen was the same as the letter that was presented two trials before. Participants indicated whether the letter did or did not match by pressing a key on the left (“q”) or right (“p”), respectively, on a standard Dutch QWERTY keyboard. Participants performed 10 practice trials and two blocks of 45 experimental trials. Reaction times on the correct trials and the number of errors served as indicators of updating performance. A trial (n) was marked as correctly encoded when the target letter (n +2) was correctly responded to. Please note that any trial is both a target trial (trial n), to be compared to a previous trial (trial n −2) as well as an encoding trial to be memorized for future comparison (on trial n +2). The percentage of matching trials was 37.8% (34 of 90).

#### Inhibition Task

To measure inhibition, we used the Stroop task. Participants responded by the use of button presses (see also Roe et al., 1980). Words were displayed in the middle of the screen one by one for 3000 ms or until a response was given. Letter font was Courier New and the font size was 40 pt. The words (oranje [orange]; blauw [blue]; groen [green]) were displayed in one of three colors (orange, RGB 205, 162, 120; blue, RGB 143, 191, 53; and green, RGB 123, 178, 208). At the bottom of the screen, the three ink colors were displayed with colored circles. Participants were instructed to press one of three designated keys on the keyboard. Colored stickers were attached to the keys. Participants had to press the button that corresponded to the ink color of the word displayed in the middle of the screen. The task consisted of three blocks of 18 inconsistent trials and 18 consistent trials each. Participants received 12 practice trials. The Stroop effect was calculated as the difference between the mean reaction times on correct incongruent and congruent trials and the difference score between errors on incongruent and congruent trials.

#### Switching Task

To measure switching, we used a Number Switch task. In this task, participants had to respond to numbers (1–10) according to different rules. When the number was printed in yellow participants indicated whether the number was odd or even. When the number was printed in blue, participants indicated whether the number was greater than five, or smaller than/equal to five (Monsell et al., 2003). Numbers were presented for 3000 ms or until a response was given. Font employed was Courier New, font size 36 pt in the colors yellow (RGB 188, 175, 81) and blue RGB, 123, 178, 208). In the first two blocks of 32 trials, participants learned the categorization rules. These two blocks required no switching because within blocks stimulus colors stayed the same (yellow or blue in counterbalanced order). The third and fourth block consisted of 32 trials each, with numbers printed in either yellow or blue, presented in random order. Hence, in these blocks, switching between the two rules was required dependent on whether colors changed (vs. stayed the same) in consecutive trials. Before the first, second and third block, participants received ten practice trials. Participants responded with a key on the left (“q”) or right (“p”) of the keyboard. Switching ability was calculated as the difference between the mean reaction times on correctly responded to switch and non-switch trials and the difference between correct responses on the switch and non-switch trials in the last two blocks.

### Data Acquisition

All stimuli were presented with experimental control software E-Prime version 2.0. Pupil diameter was recorded at 60 Hz using a Tobii T120 eye tracker, integrated into a 17-inch TFT monitor. Participants sat on a chair behind the eye tracker in a darkened room at approximately 60 cm from the screen. Data obtained from the Tobii eye tracker were processed and analyzed by the use of Brain Vision Analyzer. Custom-made macros programmed in Brain Vision Analyzer were used. The artifacts and eye blinks that were detected by the Tobii eye tracker plus three samples before and after these data points were marked as missing data. These samples were corrected using linear interpolation.

### Behavioral Data Preparation

For the 2-Back task and the Number Switch task we removed reaction times below 300 ms (0% of all trials, see e.g., Pronk et al., 2011). Given the brief response window within the 2-Back task, we did not have to correct for slow responses. For the Stroop and the Number Switch task, we excluded reaction time data that deviated more than three SDs from the mean (1.9% for congruent Stroop trials; 1.6% for incongruent Stroop trials; 0.3% for switch trials; and 0.4% for non-switch trials). For each of the three cognitive control measures, we also checked for between-subjects outliers. Participants with a performance deviating more than 3 SDs from the mean were removed from the analyses (*N* = 1 for errors on the 2-Back task;* N* = 1 for reaction times on the 2-Back task). In order to optimize power, participants were only excluded for the specific measures that concerned their extreme score, but were included in the analyses for measures for which these extreme scores were irrelevant. Errors and reaction times were checked for normality. Performance on the 2-Back task in terms of errors was skewed to the right (i.e., the mass of the distribution of the errors is concentrated on the left) and we therefore applied a square root transformation. This transformation was effective in normalizing the data distribution.

### Eye Track Data Preparation

For all tasks, we calculated pupil dilation by correcting the pupil size data for baseline pupil diameter by subtracting the average pupil diameter that was recorded during 500 ms before stimulus onset. This was done for all conditions and each participant separately. For each of the tasks we plotted the pupillary waveforms averaged across participants. Paired *t*-test were performed on these waveforms to test for the effects of trial difficulty (see below). Following visual inspection of these pupillary waveforms we also exported a 500-ms interval for each task separately as a summary measure of pupil dilation. The following criteria were used to determine the time windows used for this interval: (1) the window should include the peak in pupil dilation and (2) the interval should be (numerically) maximally sensitive to differences introduces by trial difficulty (in the Stroop and Number Switch task). The following intervals were used: for the 2-Back task: 1100–1600 ms, for the Stroop task: 1000–1500 ms, for the Number Switch task: 1300–1800 ms.

As done in earlier work (van Steenbergen and Band, 2013), mean values during the baseline and the dilation intervals were then exported to SPSS, where trials potentially affected by unreliable interpolation due to excess proportion of interpolated data (more than 70% data points interpolated in the baseline interval and/or the interval of interest) were excluded from subsequent analyses. Importantly, data points were interpolated if the Tobii eyetracker marked the left and/or right eye as missing; in addition the three samples before and after these data points were also marked to be interpolated, resulting in a quite conservative estimate of missing data. Earlier work from our lab using the same Tobii eye tracker has shown that this approach allows us to obtain the best balance between the impact of data loss (due to the exclusion of too much trials for some participants) and artifact inclusion (due to the inclusion of trials that might contain artifacts that were not reliably interpolated). Indeed, using this criterion, across tasks, on average 73% of the original number of trials were kept for analyses. The following proportions of correct trials [average across subjects (minimum—maximum)] are included for the three tasks: 2-Back task: all trials 77.6% (41.4–95.0%); Stroop task: congruent trials 78.7% (40.7–94.4%), incongruent trials 77.0% (37.7–96.2%), difference not significant (*p* = 0.168); Number-Switch task: no-switch trials 76.7% (37.9–100.0%), switch-trials: 78.3% (44.8–96.3%), difference not significant (*p* = 0.406). Please note that trial difficulty effects were also observed when using a more stringent criterion for unreliable interpolation (less than 50% interpolated data obtained in the baseline interval and/or the interval of interest). However since this criterion would exclude too many trials for some participants, this method was not suitable to obtain reliable indices of individual differences in resource allocation.

### Procedure

Participants first signed an informed consent form after which eye tracker calibration took place. Calibration was repeated in case of eye tracking problems. Participants then completed the three cognitive control tasks in a fixed order: first the Stroop task, secondly the 2-Back task, and thirdly the Number Switch task.^1^

### Analyses

Several analyses were performed to test our hypotheses concerning: (1) general individual differences in resource allocation, both between and within level of difficulty; (2) the effects of trial difficulty; and (3) error related resource allocation. In order to test effects of trial difficulty on pupil dilation, series of paired *t*-tests were performed on baseline-corrected pupil waveforms for each time point following stimulus onset, separately for the Stroop task (incongruent vs. congruent) and the Number Switch task (switch vs. no-switch task). To control for multiple comparions, trial difficulty effects were only considered significant for timepoints that exceed *p* < 0.001 and when the clusters of timepoints based on this threshold contained at least six contiguous samples. For subsequent pupil dilation analyses, we used the summary measure that extracted mean pupil dilation in the interval of interest. First, correlations across participants were obtained between behavioral data and pupil data for each cognitive control task. Second, because multiple regression analyses with pupil dilation measures as predictor variables were often compromised due to extremely high inter-predictor correlations (causing multi-collinearity problems), these correlations were inspected to investigate the predictive value of pupil dilation for performance. Only in those cases in which there are no high inter-predictor correlations, stepwise multiple regressions were used to establish a parsimonious prediction model. Furthermore, repeated measures ANOVA’s were used to test whether pupil dilation differed as a function of incorrect vs. correct responses.

## Results

**Figure 1:**
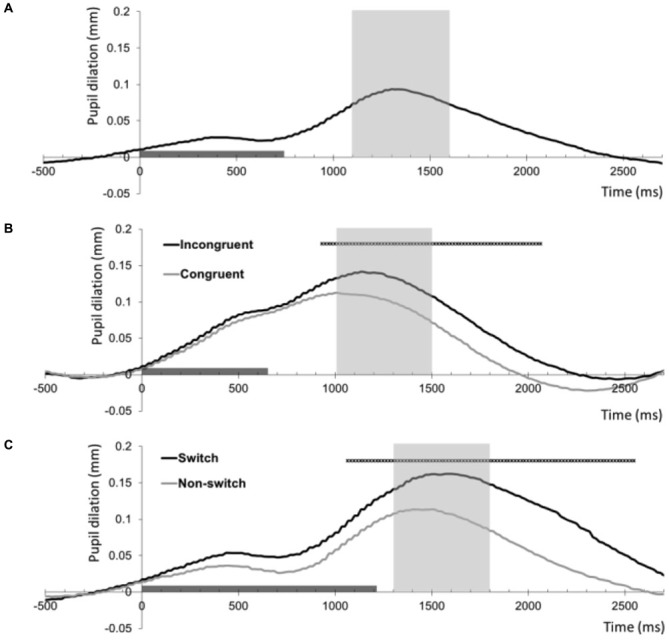
**Baseline-corrected pupil dilation (mm) averaged across all 35 participants for the three tasks as a function of time (ms)**. The timeline marker indicates the presentation of the imperative stimulus (stimulus offset shows the mean reaction time across participants). The shaded area around the peak shows the interval that was used as summary measure of mean pupil dilation. **(A)** Pupil dilation relative to the onset of target trials of the 2-Back task. **(B)** Pupil dilation relative to the onset of correct congruent and incongruent trials of the Stroop task. The black straight line indicates samples with a significant difference between these conditions (thresholded at *p* < 0.001, >5 contiguous samples). **(C)** Pupil dilation relative to the onset of correct non-switch and switch trials of the Number Switch task. The black straight line indicates samples with a significant difference between these conditions (thresholded at *p* < 0.001, >5 contiguous samples).

Means and standard deviations of the number of errors, reaction times and pupil dilation for each of the cognitive control tasks can be found in Table 1. Figure 1 shows the baseline-corrected pupillary waveforms averaged across participants. For each of the three tasks 500-ms intervals were used to analyze individual mean pupil dilation (see “Materials and Methods” Section). Analyses of the three tasks will be discussed in turn.

**Table 1:** **Descriptives for behavioral and pupillary responses, *N* = 35**.

	Behavioral data				Pupil data			
	Percentage of errors		Reaction time on correct trials (ms)		Pupil dilation on correct trials (mm)		Number of trials included in analysis	
	Mean	SD	Mean	SD	Mean	SD	Mean	Range
2-Back task	14.3	13.2	742	112	0.098	0.080	45	24–58
Stroop	3.1	2.9	664	75	0.130	0.069	81	45–99
Incongruent	4.3	4.5	705	87	0.152	0.081	40	20–51
Congruent	1.9	2.7	623	67	0.110	0.065	42	22–51
Number switch task	9.9	7.8	1195	175	0.151	0.096	45	24–58
Switch trials	10.5	8.8	1279	209	0.171	0.100	22	13–29
Non-switch trials	9.3	8.5	1110	179	0.131	0.102	23	11–31

### Pupil Dynamics in the 2-Back Task

As all trials are equally difficult in the 2-Back task, the task does not allow for a test of the relation between trial difficulty and pupil dilation. However, it provides an opportunity to test whether individual differences in pupil dilation during the task are positively correlated with performance. After analyzing the relationship between pupil dilation on correct responses and performance on the 2-Back task (in terms of errors and RT), the relationship between making errors and pupil dilation was examined.

#### Individual Differences in Resource Allocation: Effects on Error Rates

Inspection of the correlations across participants displayed in Table 2 revealed that mean pupil dilation during correct and incorrect target trials, and mean pupil dilation during correctly encoded trials were all significantly negatively related to the total number of errors on the 2-Back task, see Table 2. Most trials in the 2-Back task were either correctly responded to target trials (say, at trial n) and correctly encoded trials (at trial n −2), as only 13% of all responses were incorrect. Extremely high correlations were found between some of these measures, especially between pupil dilation on correct target trials and correctly encoded trials (i.e., *r* = 0.95, see Table 2). These high correlations would entail severe multi-collinearity problems, because unique contributions of predictors (that is, predictors’ relationship to the criterion after partialling out their correlations with other predictors) would hardly be discernable. For this reason, we refrained from subjecting these data to multiple regression analysis, and focused on the interpretation of correlations.

**Table 2:** **Correlations across participants for the 2-Back task**.

	1	2	3	4	5^a^	6^a^
1. 2-Back errors	–
2. 2-Back RT^a^	0.25	–
3. PD correct target trials	−0.51**	0.02	–
4. PD encoding of correct trials	−0.49**	−0.01	0.95***	–
5. PD incorrect target trials^a^	−0.47**	−0.10	0.64***	0.65***	–	
6. PD encoding of incorrect trials^a^	−0.16	0.06	0.45**	0.44*	0.29	–

N = 34 unless noted; PD, Pupil Dilation; *p < 0.05; **p < 0.01; ***p < 0.001; ^a^N = 33.

With regard to the number of errors in the 2-Back task, the best single predictor was pupil dilation during correctly responding to a target trial (say, at trial p). The performance on the 2-Back task was moderately highly correlated (*r* = −0.51) with this measure of pupil dilation. Participants with high pupil dilation while correctly responding made fewer errors overall than participants with relatively low pupil dilation while correctly responding. It’s worth noting that pupil dilation on correctly encoded trials (say, at trial p −2) had a virtually equal correlation with total number of errors, that is, *r* = −0.49. As explained above, the inter-correlation between these two predictors was exceptionally high (*r* = 0.95). Importantly, pupil dilation on incorrect target trials also showed a substantial negative correlation with overall error rate (*r* = −0.47), a finding that will be separately reported under the heading “Error-related pupil dilation” in “Error related pupil dilation” Section.

**Figure 2:**
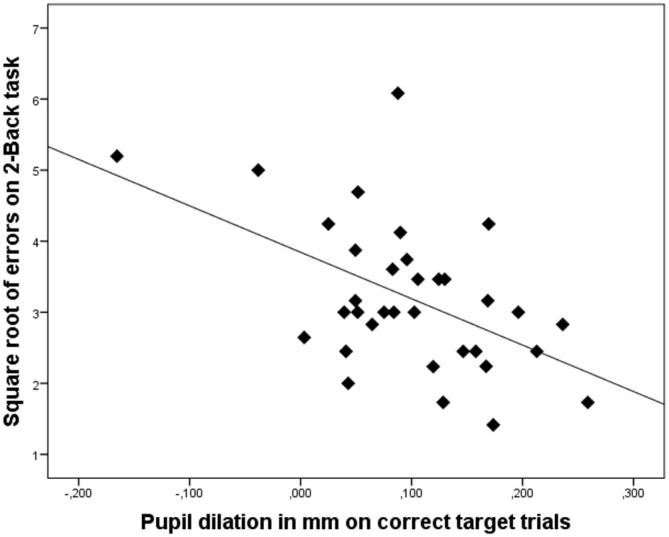
**Scatterplot illustrating that increased pupil dilation (in mm) on correct target trials is associated with better performance on the 2-Back task (lower number of errors)**. Dots represent data of individual participants.

The nature of the relation between 2-Back performance and pupil dilation on the correct trials is illustrated in the scatterplot displayed in Figure 2. These results underscore our hypothesis that on cognitively complex tasks, enhanced pupil dilation is associated with enhanced performance.

#### Individual Differences in Resource Allocation within Levels of Difficulty: Effects on Reaction Times

None of the predictor variables was significantly correlated with *reaction times* on the 2-Back task (correlations range from −0.10 to 0.06; see Table 2). Thus, it appears that pupil dilation during correct or incorrect target trials, and correctly or incorrectly encoded trials, was not related to performance concerning the speed of reaction in the 2-Back task.

#### Error Related Pupil Dilation

We subsequently explored pupil dilation during errors. Pupil dilation was marginally significantly larger for incorrect responses (*M* = 0.126, *SD* = 0.116) than for correct responses (*M* = 0.096; *SD* = 0.084), *F*_(1,33)_ = 3.77, *p* = 0.061, ${\text{η}}_{p}^{2}$ = 0.103. Furthermore, error-related pupil dilation was significantly related to fewer errors, β = −0.47, *t*_(31)_ = −2.99, *p* = 0.005, *r*^2^ = 0.22, that is, participants who displayed more error-related pupil dilation made fewer errors in the 2-Back task. Finally, higher error-related pupil dilation was not related to reaction times on the 2-Back task, see also Table 2.

### Pupil Dynamics in the Stroop Task

The Stroop task consisted of incongruent and congruent trials, of which the former are more difficult than the latter. Indeed analyses indicate that more errors were made on incongruent than on congruent trials, *F*_(1,34)_ = 9.63, *p* = 0.004, ${\text{η}}_{p}^{2}$ = 0.54, and that reaction times were slower on incongruent than on congruent trials, *F*_(1,34)_ = 147.84, *p* < 0.001, ${\text{η}}_{p}^{2}$ = 0.78. Therefore, the Stroop task enabled us to test our hypothesis concerning the relation between trial difficulty and pupil dilation. Secondly, we focus on the link between errors and pupil dilation. Thirdly, we also tested our hypotheses concerning individual differences in resource allocation for incongruent and congruent trials separately.

#### Pupillary Responses as a Function of Trial Difficulty

In line with our hypothesis, series of paired *t*-tests showed that pupil dilation was larger on correct incongruent trials than on correct congruent trials in the Stroop task As Figure 1B shows, trial difficulty increased pupil dilation in an interval ranging from 933 to 2066 ms following stimulus onset (thresholded at *p* < 0.001, >5 contiguous samples). The summary measure of peak dilation in the marked interval was used for subsequent analyses.

**Figure 3:**
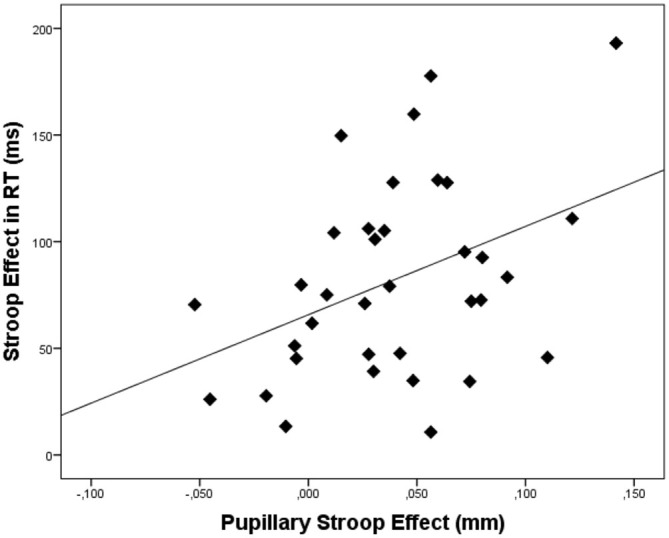
**Scatterplot depicting the relation between the pupillary Stroop effect (i.e., the difference in pupil dilation between correct incongruent and correct congruent trials) and the Stroop effect (i.e., the difference in reaction times between correct incongruent and correct congruent trials)**. Dots represent data of individual participants.

In order to link pupil dilation as a function of trial difficulty to performance on these trials, we performed regression analysis to test the relation between the pupillary Stroop effect (i.e., the difference in pupil dilation between correct incongruent and correct congruent trials) and the Stroop effect in terms of reaction times (the difference in reaction times between correct incongruent and correct congruent trials) and Stroop effect in terms of errors (the difference in the number of errors between incongruent and congruent trials). Correlations across participants can be found in Table 3. As hypothesized, the results indicated that the pupillary Stroop effect predicted the Stroop effect in terms of reaction times, β = 0.40, *t*_(33)_ = 2.49, *p* = 0.018, *r*^2^ = 0.16. Thus, the difference in pupil dilation between incongruent and congruent trials was positively related to the difference in reaction time between incongruent and congruent trials. This relation is illustrated in Figure 3. Together, these results support our hypothesis concerning the relation between effort allocation and trial difficulty.

**Table 3:** **Correlations across participants for the Stroop task**.

	1	2	3	4	5	6	7
1. Stroop effect (RT)	–
2. Stroop effect (errors)	0.20	–
3. Stroop total errors	0.12	0.47**	–
4. Stroop effect (PD)	0.40*	0.05	0.01	–
5. PD correct congruent trials	−0.20	−0.38*	−0.01	0.08	–
6. PD correct incongruent trials	0.06	−0.28	−0.00	0.61***	0.84***	–
7. PD incorrect incongruent trials^a^	−0.53**	−0.22	−0.13	0.25	0.39*	0.44*	–

N = 35 unless noted; PD, Pupil Dilation; *p < 0.05; **p < 0.01; ***p < 0.001; ^a^N = 27.

#### Error-Related Pupil Dilation

Please note that for congruent trials, the low frequency of errors precluded a statistically meaningful comparison between correct and incorrect responses. Also, because some subjects did not make any errors at all, the error analysis on incongruent trials was run with 27 subjects. As concerns incongruent trials, mean pupil dilation was larger for incorrect responses (*M* = 0.298, *SD* = 0.168) than for correct responses (*M* = 0.142; *SD* = 0.083), *F*_(1,26)_ = 28.92, *p* < 0.001, ${\text{η}}_{p}^{2}$ = 0.53.

Subsequently, we investigated error-related pupil dilation as predictor of the Stroop interference effect. We performed stepwise regression analysis including pupil dilation on correct responses and pupil dilation on incorrect responses as predictors with the Stroop effect (that is, the difference between the mean RTs of correct responses on incongruent and congruent trials) as dependent variable. Importantly, however, the relative frequency of incorrect responses is confounded by trial type. That is, the number of correct responses was higher on congruent trials than on incongruent trials, whereas—conversely—the number of errors was higher on incongruent trials than on congruent trials. Therefore, error-related pupil dilation on correct and incorrect responses combined could merely reflect the already established effect of differential pupil dilation on incongruent and congruent trials. Again, the number of errors on congruent trials was too low to be used as reliable predictor. We therefore used only correct and incorrect responses within incongruent trials and correct responses within congruent trials as predictors.

Thus, we tested whether error-related pupil dilation during incongruent trials was related to performance on the Stroop task. We performed stepwise regression analysis including mean pupil dilation during correct incongruent trials, mean pupil dilation during incorrect incongruent trials and mean pupil dilation during correct congruent trials^2^. Error-related pupil dilation during incorrect incongruent trials negatively predicted the Stroop interference effect, β = −0.53, *t*_(25)_ = −3.16, *p* = 0.004, *r*^2^ = 0.29. Thus, higher pupil dilation during error responses to incongruent trials was related to a smaller Stroop interference effect in terms of reaction times. Including pupil dilation during correct responses to incongruent trials or correct responses to congruent trials on the second step in the regression did not significantly improve the model (*p* > 0.10) and accordingly, these predictors were not found to be significantly related to the Stroop effect, β = 0.21, *t*_(25)_ = 1.14, *p* = 0.27 and β = −0.075, *t*_(25)_ = −0.40, *p* = 0.69 respectively.

#### Individual Differences in Resource Allocation

We hypothesized that enhanced resource allocation (as reflected in pupil dilation) to trials of the same difficulty level would enhance performance on these trials. To test this hypothesis, we analyzed the relation between pupil dilation and reaction times for congruent and incongruent trials separately. For incongruent trials we did not observe a significant relationship, β = −0.19, *t*_(33)_ = −1.081, *p* = 0.29, *r*^2^ = 0.03. However, on the congruent trials the relation between pupil dilation and reaction times was clearly stronger and marginally significant, β = −0.32, *t*_(33)_ = −1.963, *p* = 0.058, *r*^2^ = 0.11. In general, these results suggest that participants with higher pupil dilation tend to respond faster to Stroop trials, particularly to congruent ones.

We also analyzed the relation between pupil dilation on correct incongruent trials and the number of errors on incongruent trials and found no significant relationship, β = −0.15, *t*_(33)_ = −0.844, *p* = 0.41, *r*^2^ = 0.02.

Furthermore, we found that the Stroop interference effect in terms of errors (difference between errors on inconsistent trials and errors on consistent trials), was significantly predicted by pupil dilation on correct congruent Stroop trials (β = −0.38, *t*_(33)_ = −2.346, *p* = 0.025, *r*^2^ = 0.14).

### Pupil Dynamics in the Number Switch Task

Like the Stroop task, the Switch task also comprised relatively difficult (switch) and relatively easy (non-switch) trials, allowing for a test of the hypothesis concerning the relation between trial difficulty and pupil dilation. Indeed, analyses indicate that more errors were made in switch than on non-switch trials, *F*_(1,34)_ = 57.14, *p* < 0.001, ${\text{η}}_{p}^{2}$ = 0.63, and that reaction times were slower in switch than on non-switch trials, *F*_(1,34)_ = 33.83, *p* < 0.001, ${\text{η}}_{p}^{2}$ = 0.50. We subsequently tested hypotheses concerning error related pupil dilation and individual differences in resource allocation within the levels of task difficulty.

#### Pupil Dilation and Trial Difficulty

In line with our hypothesis concerning the relationship between trial difficulty and resource allocation, series of paired *t*-tests showed that pupil dilation was larger on correct switch trials than on correct non-switch trials in the Number Switch task as Figure 1C shows, trial difficulty increased pupil dilation in an interval ranging from 1066 to 2550 ms following stimulus onset (thresholded at *p* < 0.001, >5 contiguous samples). The summary measure of peak dilation in the marked interval was used for subsequent analyses.

We first tested whether the difference in pupil dilation between correct switch trials and correct non-switch trials predicted overall performance on the switch task. Correlations across participants can be found in Table 4. Results indicated that the difference in pupil dilation between correct switch and correct non-switch trials was not predictive for switch costs in terms of errors (the difference in the number of errors between switch and non-switch trials) nor for switch costs in terms of reaction times (the difference in reaction times between correct switch and correct non-switch trials), see also Table 4.

**Table 4:** **Correlations across participants for the Number Switch task**.

	1	2	3	4	5	6	7
1. Switch cost correct	–
2. Switch cost RT	−0.02	–
3. Errors switch task	−0.03	0.35*	–
4. PD switch minus
non-switch	0.19	0.10	−0.15	–
5. PD correct
non-switch trials	−0.14	0.14	−0.09	−0.35*	–
6. PD correct switch
trials	−0.02	0.21	−0.18	0.29	0.81***	–
7. PD incorrect switch
trials^a^	0.01	−0.08	−0.52**	0.33	0.09	0.30^†^	–

N = 35 unless noted; PD, Pupil Dilation; ^†^*p* < 0.10; *p < 0.05; **p < 0.01; ***p < 0.001; ^a^N = 31.

#### Error-Related Pupil Dilation

Due to the low frequency of errors on non-switch trials, a reliable measure of error-related pupil dilation on non-switch trials was not feasible. In addition, because some subjects did not make any errors at all, the error-related pupil dilation analysis for switch trials was run with 31 subjects.

Pupil dilation was larger for incorrect switch responses in switch trials (*M* = 0.245, *SD* = 0.189) than for correct responses in switch trials (*M* = 0.172; *SD* = 0.109), *F*_(1,30)_ = 4.74, *p* = 0.037, ${\text{η}}_{p}^{2}$ = 0.14.

Subsequently, we tested whether error-related pupil dilation during switch trials was related to performance on the Number Switch task. We performed stepwise regression analysis, with pupil dilation during correct switch trials, pupil dilation during incorrect switch trials and pupil dilation during correct non-switch trials as predictors and switch costs as dependent variable (separately in terms of RT and errors). Pupil dilation in both incorrect and correct switch trials as well as pupil dilation in correct non switch trials appeared to be unrelated to switch cost both in terms of errors and reaction times, see Table 4. Thus, higher pupil dilation during an error was not related to better switch performance.

Apart from switch costs, we also investigated pupil dilation as a predictor of the total number of errors as dependent variable. Because errors on non-switch trials occurred too infrequently, we again only analyzed pupil dilation during correct non switch trials and correct and incorrect switch trials. Stepwise regression analysis indicated that higher error-related pupil dilation in the switch trials was related to fewer errors in the Number Switch task, β = −0.52, *t*_(31)_ = −3.35, *p* = 0.002, *r*^2^ = 0.27. The other predictors were unrelated to the dependent variables (see Table 4) and did not significantly improve the model (all *p* > 0.10).

#### Individual Differences in Resource Allocation

In order to test the hypothesis that enhanced resource allocation to trials with the same difficulty level enhances performance on these trials, we analyzed the relation between pupil dilation on correct responses and the reaction time on these trials for switch trials and non-switch trials separately. We did not observe a relation between pupil dilation on correct switch trials and reaction times to these trials, β = 0.18, *t*_(33)_ = 1.065, *p* = 0.30, *r*^2^ = 0.03. Similarly, no such relation was found using the number of errors in switch trials as dependent variable, β = −0.18, *t*_(33)_ = 1.044, *p* = 0.30, *r*^2^ = 0.03. Similar analyses were conducted on the non-switch trials, revealing no significant effects, both *p*’s > 0.70. In sum, within the Number Switch task, no evidence was obtained for the hypothesis concerning the relation between individual differences in resource allocation and performance.

## Discussion

Using pupillometry, the present research aimed to investigate the role of resource allocation during updating, inhibition and switching tasks. Investigating resource allocation in tasks tapping into the three cognitive control components provides useful insight in the unique characteristics of these tasks. This study is the first to investigate resource allocation by means of pupil dilation in all three cognitive control components. In the current research we focused on three potential sources of pupil dilation during the cognitive control tasks, notably individual differences, trial difficulty, and the commitment of errors. We will review and discuss our findings on the basis of these three sources of resource allocation.

### Individual Differences in Resource Allocation

Our first hypothesis related to the idea that enhanced resource allocation would enhance performance in cognitively complex tasks. We found strong evidence for this hypothesis in the 2-Back task (measuring updating). Responding correctly to trial p in the 2-Back task requires correct retrieval of the identity of the stimulus on trial p −2, which in turn is virtually completely dependent on the correct encoding of the stimulus identity on trial p −2. Consistent with this idea we found that both pupil dilation in correct target trials (p) and pupil dilation in correct encoding trials (p −2) were substantially interrelated and both these pupillary measures were strong predictors of overall performance on the 2-Back task. Thus, performance on the 2-Back task benefits from consistent allocation of resources to trials. With consistent resource allocation, we mean here, and in subsequent text, that resources must be allocated immediately upon stimulus presentation for each stimulus, to enable adequate comparison with previously presented stimuli and to ensure proper encoding for future comparison. Only when a stimulus is encoded correctly *and* the target letter is correctly compared to the encoded stimulus, performance on the 2-Back task is successful.

Note that the strongly inter-related high correlations of pupil dilation at trials p −2 (encoding) and p (responding) correlation with overall performance in terms of low error rates was based on between-subjects correlations. This pattern can be interpreted as possibly reflecting the fact that participants who consistently allocate relatively much resources to the processing of successive trials (i.e., to properly encode them for use at p +2, and to compare them correctly with p −2) show a better performance on the task as a whole (fewer errors) than participants who allocate relatively fewer resources to processing the trials or do so less consistently.

The finding that pupil dilation was related to performance in the updating task that was used in the present study, namely a 2-Back task, is not completely in line with the findings from Karatekin et al. (2007, 2009), where no relation was found between pupil dilation and overall performance for healthy controls on an n-Back task. However, the n-Back tasks they used in their studies, a spatial 0- and 1-Back task, were probably quite easy for healthy controls, and therefore required fewer resources. We showed that for a more difficult version of the n-Back task, namely a 2-Back task, pupil dilation was indeed predictive for performance on the 2-Back task. These findings support the idea that only when a task is cognitively demanding, resource allocation, as measured by pupil dilation, is related to overall performance (see for example van der Meer et al., 2010). The present findings provide more insight in the characteristic features of an updating task. Failure to focus on a stimulus in an updating task has consequences for both the ability to indicate whether the stimulus is the same as the stimulus presented n trials ago, as for the ability to correctly encode the stimulus, which is needed to perform correctly on n trials later.

Although the effect was clearly pronounced in the updating task, we found a trend towards a relation between pupil dilation within the Stroop task (on correct trials) and speed of responding to trials. When time pressure is high and people are trying to respond as fast as possible, even a simple task as responding to consistent Stroop trials may be enhanced by resource allocation. At the same time, we have to interpret these effects with some caution, because the effect was only marginally significant and the relation was not obtained for inconsistent trials (although it was in the right direction). It is interesting to think about the overarching processes that help people to consistently allocate resources to a task. One possibility is the role of working memory in these tasks.

We also found pupil dilation on correct congruent trials to be negatively related to the Stroop effect in terms of errors. That is, participants with higher pupil dilation on congruent trials (virtually always responded to correctly) had a weaker Stroop effect—that is showed a smaller difference between errors on incongruent trials than errors on congruent trials. This effect is somewhat enigmatic because one would expect higher pupil dilation on congruent trials to be associated with fewer errors on *congruent* trials which—everything else being equal—would result in a stronger Stroop effect, not a weaker one. On a speculative note, an alternative account might be that pupil dilation on relatively easy (congruent) trials is a more diagnostic indicator of overall task motivation than pupil dilation on relatively difficult (incongruent) trials, because the latter might autonomously trigger higher pupil dilation in participants irrespective of overall task motivation. In other words, participants who allocate effort even to the easy trails must be really motivated compared to participants who don’t. This way, paradoxically, pupil dilation on easy trials (almost always correctly responded to) may turn out the best indicator of overall task performance in terms of the error difference between easy and difficult trials.

### Trial Difficulty

A second factor related to resource allocation in cognitive control tasks is *trial difficulty*. In the n-back task the difficulty of the trials remained constant across the task which disqualifies this task for testing this hypothesis. However, in both the Stroop task (consistent vs. inconsistent trials) and the Switch task (switch vs. non-switch trials) the results were generally in line with our hypothesis. In the Stroop task, incongruent trials were related to higher pupil dilation as compared to congruent trials, indicating that incongruent trials involve conflict and thus elicit more resource allocation. Similarly, in the switching task, we found that switch trials elicited higher pupil dilation compared to non-switch trials (i.e., trials on which the same response rule had to be applied as on the previous trial), indicating that the former require more resource allocation than the latter.

Moreover, the results showed that differences in pupil dilation as a function of task difficulty were also related to differences in performance. We replicated the results from Laeng et al. (2011), who found that a higher pupillary Stroop was related to a stronger Stroop interference effect in terms of reaction times. These results are most easily explained by the idea that people recruit more resources to overcome the difficulty in the trial. However, at the same time, with increasing levels of difficulty, performance will decrease. In a way, then, difficult trials may evoke more resources than easy trials, but still not sufficiently so to compensate for performance differences. Although, pupil dilation as a function of trial difficulty was also obtained in the Switch task, unlike the Stroop task, this difference in pupil dilation between switch vs. non-switch trials was unrelated to performance. It could be the case that for switch trials compared to non-switch trials, the enhanced resource allocation does to some extent compensate for difficulty.

### Error-Related Pupil Dilation

A third source of pupil dilation in the cognitive control tasks relates to making errors. In all tasks we explored pupil dilation on incorrect trials (vs. correct trials). We found that pupil dilation was larger for incorrect responses as compared to correct responses in the inhibition and switching task, but not in the updating task. This finding replicates other studies that have observed such error-induced increases in physiological arousal, as measured by pupil dilation (Critchley et al., 2005), skin conductance response, and heart rate deceleration (Hajcak et al., 2003, 2004; O’Connell et al., 2007). The absence of this effect in the updating task might be due to the fact that more errors were made in this task as compared to the inhibition and switching task, or that people might be less likely to register the errors they make.

The data from the Stroop task indicated that individuals with higher pupil dilation during errors proved to be more effective in inhibition as indicated by a decreased Stroop interference effect in terms of reaction times (measured during correct trials). Based on these preliminary data, it might be speculated that error-related pupil dilation serves as an index for the activity of a neurocognitive system that plays a role in error detection as well as the optimization of inhibitory control (see also Larson and Clayson, 2011). A possible candidate is the anterior cingulate cortex, a region which drives autonomic arousal (Critchley et al., 2005), monitors actions and errors (Gehring et al., 1990; Holroyd and Coles, 2002) and adaptively regulates cognitive control (see e.g., Posner et al., 1988).

### Similarities and Differences in Resource Allocation Between Tasks

The findings from the present study highlight the diversity of the three cognitive control components in terms of resource allocation. Pupil dilation in the inhibition task and the switching task were strongly related to the characteristics of the presented stimuli, that is, the difficulty of the trials, and of the responses given, specifically the errors committed. In the updating task, the presented stimuli did not differ in difficulty, but it was found that the higher the pupil dilation at both encoding of stimuli and responding to target stimuli, the better the performance on the task. In sum, findings concerning both the inhibition task and the switching task show that pupil dilation in these tasks seems to reflect specific features of the presented stimuli whereas the observed pupil dilation in the updating task seem to reflect consistent focus on the task.

The consistent focus on the task as obtained in the updating task might be closely related to the idea of goal activation: the ability to keep the task requirements active in mind throughout the whole task (Duncan et al., 1997). Miyake et al. (2000) suggested that keeping goal-relevant and task-relevant information in mind might be a common task requirement of all cognitive control tasks. This is in line with the idea from Nieuwenhuis et al. (2004), who suggested that the ability to turn task requirements into active goals and maintain these goals throughout the task is a key component of cognitive control. The findings concerning the relation between resource allocation and the performance on the updating task fit in here. For this task, consistent monitoring of the stimulus is crucial for performance, measuring the ability to activate goals and maintain these goals active throughout the task. The link between pupil dilation and general speed of responding in the Stroop task may also relate to goal maintenance; if one consistently acts on the goal to react as fast as possible, enhanced resource allocation support this goal directed action. As we indicated before, given the relative weak results for the latter effects, caution and replication seem warranted here.

Consistent resource allocation during the inhibition and switching task seems less crucial for overall performance as in the updating task, which is in line with the constructs these tasks are trying to tap into. In the inhibition and switching task, one can temporarily experience decreased goal activation during a specific stimulus. That is, the performance on a later trial is not contingent on the performance on a previous trial. Instead, for the updating task, decreased goal activation during one stimulus has consequences for performance on following trials. This is exactly what updating grasps: the ability to *constantly* monitor incoming information.

### Limitations and Future Research

The findings from the present research provide useful insight in the unique characteristics of the three cognitive control components updating, inhibition and switching in terms of resource allocation. Yet, some limitations should be pointed out. First, the nature of our design was correlational. Therefore, although theoretically we consider pupil dilation as an index for effort allocation, which subsequently may causally influence performance, the reverse causal direction (performance influencing pupil dilation) remains possible. Second, the present research only used a selection of cognitive control tasks tapping into updating, inhibition and switching. Future research should investigate whether the observed pupil dynamics in the three administered cognitive control tasks are similar to those found in other cognitive control tasks, such as for example a number-letter task, a go/no-go task or a keep track task. Furthermore, it would be of interest to investigate how adaptive resource allocation is related to processes that are assumed to be central for goal maintenance and updating, such as working memory processes (cf. Miyake and Friedman, 2012).

Second, although the present findings provide some insight in error-related pupil dilation for the cognitive control tasks we used, a more thorough investigation is needed to draw firm conclusions about the neural mechanisms underlying error-related resource allocation in updating, switching and inhibition tasks. Although there is accumulating evidence for a link between error-related pupil dilation and neural activity in the anterior cingulate (Critchley et al., 2005; Murphy et al., 2014; Ebitz and Platt, 2015), future research is needed to show how error-related pupil dilation relates to the particular cognitive components involved in error processing, for example using event-related brain potential (ERP) methods (Wessel et al., 2011).

Finally, an a more general note, the current research set-up did not allow to test the role of the brain regions that are involved in the relation between pupil dilation and executive functioning. Since pupil dilation might reflect activity of the LC-NE system (Aston-Jones and Cohen, 2005), this system might be speculated to differentially modulate the temporal dynamics of the neural structures supporting executive functioning (see e.g., Miyake and Friedman, 2012). We hope that our results inspire future neuroscientific studies to get a better understanding of the role of the LC-NE system in the neural mechanisms underlying EF.

## Conclusion

The present study aimed at getting more insight in when and how people allocate resources during updating, inhibition and switching tasks by focusing on three sources of pupil dilation. First, we focused on individual differences in consistent resource allocation. The results suggest that performance on an updating task, but not an inhibition or switching task, is highly dependent on consistently allocating effort. Second, results indicated that trial difficulty strongly influenced the allocation of resources in both the inhibition task and the switching task. However, only for the inhibition task this effect was related to overall performance. Third, the results also provide preliminary evidence that error-related pupil dilation might not be a shared mechanism among the three cognitive control tasks that were used in the present study. In sum, by systematically linking the three cognitive control components to processes of resource allocation, we were able to highlight similarities and diversity between the components that sustain goal-directed action.

## Conflict of Interest Statement

The authors declare that the research was conducted in the absence of any commercial or financial relationships that could be construed as a potential conflict of interest.

## Footnotes

^1^We also measured pupil dilation during the Wisconsin Card Sorting Task (administered before the Stroop task) and a strategy game (administered after the Number Switch task). After this strategy game, participants completed the Raven. Reporting the results concerning these tasks is beyond the scope of the current article.

^2^Note that the 0.84 correlation between the predictors PD correct congruent and PD correct incongruent trials might cause multi-collinearity problems in this multiple regression. However, because the correlations of both these predictors with the dependent variable were negligible, stepwise regression was deemed appropriate.
